# Different therapeutic effects of cells derived from human amniotic membrane on premature ovarian aging depend on distinct cellular biological characteristics

**DOI:** 10.1186/s13287-017-0613-3

**Published:** 2017-07-27

**Authors:** Chenyue Ding, Hong Li, Yun Wang, Fuxin Wang, Huihua Wu, Rulei Chen, Jinghuan Lv, Wei Wang, Boxian Huang

**Affiliations:** 1 Center of Reproduction and Genetics, Suzhou Hospital Affiliated to Nanjing Medical University, Suzhou, 215002 China; 2 State Key Laboratory of Reproductive Medicine, Nanjing Medical University, Nanjing, 210029 China; 3 Department of Obstetrics and Gynecology, Suzhou Hospital Affiliated to Nanjing Medical University, Suzhou, 215002 China; 4 Department of Pathology, Suzhou Hospital Affiliated to Nanjing Medical University, Suzhou, 215002 China

**Keywords:** Premature ovarian aging, Human amniotic mesenchymal stem cells, Human amniotic epithelial cells, Cellular biological characteristics

## Abstract

**Background:**

Many reports have shown that various kinds of stem cells have the ability to recover premature ovarian aging (POA) function. Transplantation of human amniotic epithelial cells (hAECs) improves ovarian function damaged by chemotherapy in a mice model. Understanding of how to evaluate the distinct effects of adult stem cells in curing POA and how to choose stem cells in clinical application is lacking.

**Methods:**

To build a different degrees of POA model, mice were administered different doses of cyclophosphamide: light dose (70 mg/kg, 2 weeks), medium dose (70 mg/kg, 1 week; 120 mg/kg, 1 week), and high dose (120 mg/kg, 2 weeks). Enzyme-linked immunosorbent assay detected serum levels of sex hormones, and hematoxylin and eosin staining allowed follicle counting and showed the ovarian tissue structure. DiIC_18_(5)-DS was employed to label human amniotic mesenchymal stem cells (hAMSCs) and hAECs for detecting the cellular retention time in ovaries by a live imaging system. Proliferation of human ovarian granule cells (ki67, AMH, FSHR, FOXL2, and CYP19A1) and immunological rejection of human peripheral blood mononuclear cells (CD4, CD11b, CD19, and CD56) were measured by flow cytometry (fluorescence-activated cell sorting (FACS)). Distinction of cellular biological characteristics between hAECs and hAMSCs was evaluated, such as collagen secretory level (collagen I, II, III, IV, and VI), telomerase activity, pluripotent markers tested by western blot, expression level of immune molecules (HLA-ABC and HLA-DR) analyzed by FACS, and cytokines (growth factors, chemotactic factors, apoptosis factors, and inflammatory factors) measured by a protein antibody array methodology.

**Results:**

After hAMSCs and hAECs were transplanted into a different degrees of POA model, hAMSCs exerted better therapeutic activity on mouse ovarian function in the high-dose administration group, promoting the proliferation rate of ovarian granular cells from premature ovarian failure patients, but also provoking immune rejection. Meanwhile, our results showed that the biological characteristics of hAMSCs were superior to hAECs, but not to expression of immune molecules.

**Conclusions:**

These results suggest that hAMSCs are a more effective cell type to improve ovarian function than hAECs. Meanwhile, this distinct effect is attributable to cellular biological characteristics of hAMSCs (telomerase activity, expression level of pluripotent markers, cytokine and collagen secretion) that are superior to hAECs, except for immunological rejection. Sufficient consideration of cell properties is warranted to move forward to more effective clinical therapy.

**Electronic supplementary material:**

The online version of this article (doi:10.1186/s13287-017-0613-3) contains supplementary material, which is available to authorized users.

## Background

The ovary is a unique and complex organ responsible for the generation of an appropriate number of developmentally competent oocytes through folliculogenesis [1]. The major function of the ovary is to regulate female fertility and keep women healthy during a definite life stage. However, premature ovarian aging (POA) affects 1% of women in the general population [2]. A loss of oocytes and fertility potential is correlated with exposure to genetic abnormalities, pelvic irradiation, and systemic chemotherapy [3].

Ovarian reserve (OR) is widely deemed to be the sum of all remaining available follicles in ovaries. Therefore, it is a predictive criterion for the outcome of infertility treatment. Various standards have been proposed to assess OR. Estradiol and inhibin A are largely produced by the dominant and preovulatory follicle and thus largely reflect ovulatory activity [4]. The FSH level is a key factor to affect the selection of a limited number of follicles at the stage of cyclic recruitment [5, 6]. Meantime, AMH is an excellent marker for the number of small, growing follicles and is produced by the granulosa cells of follicles from the time at which follicle growth is first initiated, which might aid in the diagnosis and follow-up of women at risk of POA [7]. Consequently, the hormone level of E2, FSH, and AMH is a classical test criterion for POA. Additionally, a key point of POA is the decrease in oocyte quality as well as quantity. Therefore, the number of antral follicles and the viability of granulosa cells are regarded as the evaluation standard for POA [8].

Many research studies indicate that the most severe form of decreased ovarian reserve (DOR) is represented by premature ovarian failure (POF) in young females [9–11]. Therefore, according to the aforementioned criteria, two different degrees of POA are defined: DOR and POF. Currently, POA cannot be reversed and although treatments are available using long-term hormone replacement, the therapy relieves menopausal symptoms but cannot restore ovarian aging in women. There is an urgent need to improve treatment strategies. Regenerative medicine research suggests that due to the self-renewal capacity and multiplex differentiation potential of stem cells, they could be used to cure various human diseases [12]. Recent interest in the therapeutic potential of stem cells has grown and multipotent stem cells could be developed from different sources, such as bone marrow [13], adipose tissue [14], amniotic fluid, and the amnion [15, 16], and all have been shown to have potential to restore ovarian function and rescue long-term infertility in chemotherapy-treated female mice. However, little is still known about which kind of stem cells will exert the best therapeutic activity and how to choose the stem cells for POA, especially in clinical therapy.

The human amniotic membrane (hAM) is a kind of superior biomaterial that could be suitable for allotransplantation and regenerative medicine. hAM includes two different stem cell populations: human amniotic epithelial cells (hAECs) and human amniotic mesenchymal stem cells (hAMSCs). There are several advantages which make these suitable for clinical therapy: acquirement of a high cell number, easy to obtain because hAM is usually discarded after delivery, and their use is within the legal and ethical framework. Meanwhile, compared to adipose tissue-derived stromal cells (ADSCs) and bone marrow mesenchymal stem cells (BMSCs), stem cells derived from hAM possess characteristics of a less invasive procedure.

Although hAMSCs and hAECs are all derived from hAM, the characters of both cell types not only show similar aspects but also demonstrate distinction. Therefore, the purpose of the present study was to determine whether hAMSCs and hAECs exhibit different therapeutic potential in a mice model of ovarian aging disease and cell samples from DOR and POF patients.

## Methods

### Establishment of a mice model with different degrees of POA

Female ICR mice between 7 and 8 weeks of age were obtained from Nanjing Medical University with Institutional Animal Care and Use Committee approval in accordance with institutional guidelines. To build the mice model with different degrees of ovarian aging, different doses of CTX (Sigma, USA) were classified into three groups: light-dose treatment (70 mg/kg, 2 weeks), medium-dose treatment (70 mg/kg, 1 week; 120 mg/kg, 1 week), and high-dose treatment (120 mg/kg, 2 weeks). The animals were divided into four groups (per group, *n* = 10) respectively after CTX treatment: control group (saline injection), light-dose CTX treatment group, medium-dose CTX treatment group, and high-dose CTX treatment group. Follicle numbers, weight of the ovary, hormone level, and litter size were estimated after cell transplantation.

### Assessment of ovarian function by comparison of ovarian follicle counts and litter sizes

After cell transplantation, mice (cell translated and nontransplanted) were euthanized from 0 to 4 weeks, ovaries on both sides were removed and fixed by 10% formalin, paraffin embedded, serially sectioned at 5-mm thickness, mounted in order on glass microscope slides, and stained with hematoxylin and eosin (HE). Four stages of follicles (primordial, primary, secondary, and antral follicles) were detected and classified. The ratio of the number of follicles from the ovary on both sides at four stages was calculated and compared between each group (per group, *n* = 10). Three representative sections from each ovary were selected. Only follicles containing an oocyte were counted to avoid counting any follicle twice. To test the safety of cell transplantation and improvement in ovarian function, we compared the litter sizes obtained by natural mating after the transplantation of hAMSCs and hAECs into the ovaries on both sides. Eight weeks after cell transplantation, cell transplanted and nontransplanted model mice with different degrees of ovarian aging (per group, *n* = 10) were found to be in proestrus based on a vaginal smear test, and were then put into the same cage with male mice for natural mating at a ratio of 1:2. The number of offspring was then recorded.

### Enzyme-linked immunosorbent assay analysis

Mouse plasma was harvested to evaluate the expression level of E2, AMH, or FSH using an enzyme-linked immunosorbent assay (ELISA) kit (Mybiosource, USA) according to the manufacturer’s guide. Briefly, 50 μl of serum sample was added to each well. The test plate was wrapped with membrane, and incubated for 30 min at 37 °C. Thereafter, wells on the plate were dried and washed with Wash Buffer five times (10 s per time). Then 50 μl HRP-conjugate reagent was added into each sample well and incubated for 60 min at 37 °C. Samples were washed with Wash Buffer five times (10 s per time). Subsequently, 50 μl substrate A Solution followed by 50 μl substrate B Solution was added and incubated for 15 min at 37 °C. Then 50 μl Stop Solution was added to each control and sample well. Finally, the light absorbance was measured and recorded by a spectrophotometer (Varian Company, Australia).

### Preparation and culture of hAECs and hAMSCs

Human placentas were obtained from term pregnancy during uncomplicated caesarean sections. Written and informed consent was obtained from each woman who tested negative for HIV-I and hepatitis B and C. The institutional ethics committee approved the use of human amnions for this project. According to the protocol reported previously [17], the amniotic membrane was mechanically separated from the chorion, and cut with a razor to yield 1.5–2.0 cm^2^. The amniotic membrane segments were digested with 0.25% Trypsin/EDTA at 37 °C for 45 min to isolate hAECs. After repeating this treatment several times, the epithelial cells were removed completely. The rest of the tissue pieces were placed in DMEM containing collagenase I (1 mg/ml; Gibco, USA) and DNase I (1 mg/ml; Sigma, USA) and were incubated at 37 °C for 60 min to isolate hAMSCs. The isolated hAECs and hAMSCs were cultured with DMEM medium supplemented with 10% FBS (Gibco, USA), penicillin/streptomycin (Gibco, USA), glutamine (Gibco, USA), EGF (R&D, USA), and bFGF (R&D, USA) and incubated at 37 °C, 5% CO_2_. When cells reached 80–90% confluence, adherent cells were trypsinized and passaged.

### hAMSC and hAEC phenotypic characterization

hAMSC and hAEC specific surface antigens were stained with PE-conjugated antibodies of anti-human-CD-105, anti-human-CD-29, anti-human-CD-44, anti-human-CD-73, anti-human-CD-90, anti-human-CD-34, anti-human-CD-45, anti-human-EpCam, or anti-human-CD-166 (BD, USA) or their corresponding isotype control. The stained cells were then analyzed respectively by fluorescence-activated cell sorting (FACS). The detailed instruction is the same as the FACS analysis already described. Differentiation ability of hAMSCs was tested using hAMSC differentiation kits for culture (Thermo, USA).

### Isolation of primary human ovarian granulosa cells and human peripheral blood mononuclear cells from DOR and POF patients

Patients with tubal occlusion served as the control group. Patient selection followed a standard as follows. DOR was defined as antral follicle count < 5 or AMH < 1.1 ng/ml and FSH ≥ 10 mIU/ml. Women aged > 40 years were not included in this study in order to exclude patients with physiological ovarian aging. POF patients were recruited according to inclusion criteria that consisted of primary amenorrhea or secondary amenorrhea for at least 4 months, younger than 40 years of age, and at least two recordings of serum concentrations of FSH measurements exceeding 40 IU/L. Women with known normal karyotype, previous chemotherapy or radiotherapy, autoimmune diseases, or ovarian surgery were excluded. Primary human ovarian granulosa cells (hGCs) were obtained following informed consent from tubal occlusion (TO; *n* = 28), DOR (*n* = 31), and POF (*n* = 43) patients respectively after approval from the Suzhou Hospital Affiliated to Nanjing Medical University Research Ethics Board. All patients were treated with recombinant FSH (Puregon; Schering Plough, NJ, USA) and GnRH antagonist Ganirelix (Merck, Frosst, Montreal, Canada). Vaginal ultrasound examination was performed to monitor follicular development. Final follicular maturation was induced by administering 10,000 IU of human chorionic gonadotropin (hCG) (Pregnyl; Merck). hGCs were purified using density centrifugation from follicular aspirates collected from women undergoing oocyte retrieval as described previously [18]. Primary hGCs were cultured in six-well plates in DMEM/F12 media (Thermo, USA) containing 1% penicillin/streptomycin, 10% fetal bovine serum (complete medium), 100 mg/ml of streptomycin sulfate (Thermo, USA), and 1× GlutaMAX (Thermo, USA). The culture medium was changed every other day in all experiments. We collected human peripheral blood mononuclear cells (hPBMCs) from TO (control group, *n* = 41), DOR (*n* = 48), and POF (*n* = 58) patients in our reproductive center, and then hPBMCs were filtered for conditions such as Addison disease, autoimmune oophoritis, and few cells. Finally, hPBMCs were obtained from TO (*n* = 20), DOR (*n* = 29), and POF (*n* = 36) patients respectively after informed consent was obtained. Women with known Addison disease and autoimmune oophoritis were excluded. Venous blood was collected from healthy volunteers into BD sodium-heparin tubes. hPBMCs were separated by density-gradient centrifugation using a Percoll separation medium (PSM; Sigma, USA). In brief, 20 ml of twofold diluted peripheral blood from healthy donors were layered on 15 ml of PSM and centrifuged at 400 × *g* for 30 min at room temperature. hPBMCs were collected and were cultured in six-well plates in RPMI-1640 media (Thermo, USA).

### Live imaging of transplanted hAMSCs and hAECs in a mice model

According to the protocol instruction, probe DiIC_18_(5)-DS (Life Technologies, USA) was employed to label hAMSCs and hAECs for detection. Next, after tail-vein injection of Dil-labeled hAMSCs and hAECs from 6 h to 14 days respectively, cell transplanted mice (*n* = 15) and nontransplanted mice (*n* = 15) were screened with a live imaging system (Xenogen IVIS 100 in vivo Imaging System; PerkinElmer, USA) to characterize, quantify, and visualize Dil-labeled cells. To avoid a very weak detecting signal caused by fur, mice were anesthetized and the abdominal fur shaved which was convenient for signal detection.

### Western blot analysis

hAMSCs and hAECs from three pregnant women were harvested and dissociated in a lysis buffer. Protein was extracted from each sample, which was then loaded onto 10% gels and separated by sodium dodecyl sulfate polyacrylamide gel electrophoresis (SDS-PAGE). Next, the separated proteins were transferred to polyvinylidene difluoride membrane (PVDF; Millipore, USA). Thirdly, the proteins were incubated with the primary antibodies (Abcam, USA) of anti-human-collagen I, II, III, IV, and VI, anti-human-telomerase reverse transcriptase, anti-human-OCT4, anti-human-NANOG, anti-human-Gapdh, anti-human-β-Tubulin, anti-human-SSEA4, or anti-human-TRA-1-81 and appropriate secondary antibodies (goat anti-rabbit HRP conjugates; Jackson Immunoresearch, West Grove, PA, USA) separately. The specific signals were detected by enhanced chemiluminescence (Pierce ECL Western blotting Substrate; Thermo). Finally, the membrane was checked by a chemiluminescence detection system (Tanon, China) and the signal intensity of each band was analyzed using Imaging J Software (National Institutes of Health, USA). Experiments were repeated three times, results are presented as fold change ± SD, and *p* < 0.05 is determined as significant difference.

### FACS analysis

hAMSCs and hAECs were digested separately by Trypsin/EDTA for 3 min and blown gently into single cells, which were fixed and permeated by the Cytofix/Cytoper Fixation/Permeabilization Solution Kit (BD, USA) following the manufacturer’s instruction. Treated cells were then stained with PE-conjugated or FITC-conjugated antibodies of anti-human-ki67 (Abcam, USA), anti-human-AMH (Thermo, USA), anti-human-FSHR (Thermo, USA), anti-human-FOXL2 (Thermo, USA), anti-human-CYP19A1 (Abgent, USA), anti-human-CD8 (BD, USA), anti-human-CD4 (BD, USA), anti-human-CD11b (BD, USA), anti-human-CD19 (BD, USA), or anti-human-CD56 (BD, USA) or their corresponding isotype control, for 30 min at 4 °C as already described. The stained cells were analyzed on fluorescence-activated cell sorter (Beckman, USA). Experiments were repeated three times, results are presented as fold change ± SD, and *p* < 0.05 is determined as significant difference.

### Immunofluorescence staining

The primary antibodies of anti-human-BrdU (Abcam, USA), anti-human-AMH (Abcam, USA), anti-human-FSH (Abcam, USA), anti-human-inhibin α (Abcam, USA), and anti-human-inhibin β (Abcam, USA) were selected to characterize ovarian granular cells. For the staining procedure, ovarian sections were fixed with 4% (w/v) paraformaldehyde (PFA; Sigma, USA) at room temperature for 10 min and then washed three times for 5 min with phosphate buffer solution (PBS), permeated with 0.1% Triton X-100 (Sigma, USA)/PBS on ice for 10 min, and blocked with fresh 4% bovine serum albumin (BSA; Sigma, USA)/PBS at room temperature for 30 min. The treated cells were washed with PBS three times for 5 min and then incubated with primary antibodies overnight at 4 °C. After rinsing with PBS for 5 min, the cells were stained by Cy2-conjugated or FITC-conjugated secondary antibodies (Jackson Immunoresearch) in the dark at room temperature for 30 min. The stained cells were mounted with 4′,6-diamidino-2-phenylindole (DAPI; Vector Lab, USA) after washing with PBS for 5 min, and then photographed under a fluorescence microscope (Olympus, Japan).

### Antibody microarray analysis

Cytokines were measured by a protein antibody array methodology (RayBio Human Cytokine Antibody Array, RayBiotech G Series 2000; RayBiotech, Inc., Norgross, GA, USA) that contains antibodies targeted to detect protein expression levels which are differentially expressed in hAMSC-conditioned media (CM) and hAEC-CM. One hundred micrograms of CM was used according to the manufacturer’s instructions.

### Statistical analysis

All results were shown as means ± SD. Statistically significant difference was determined by one-way ANOVA with SPSS 17.0 software, and *p* < 0.05 was regarded as statistical significance.

## Results

### Established mice model with different levels of POA

**Fig. 1:**
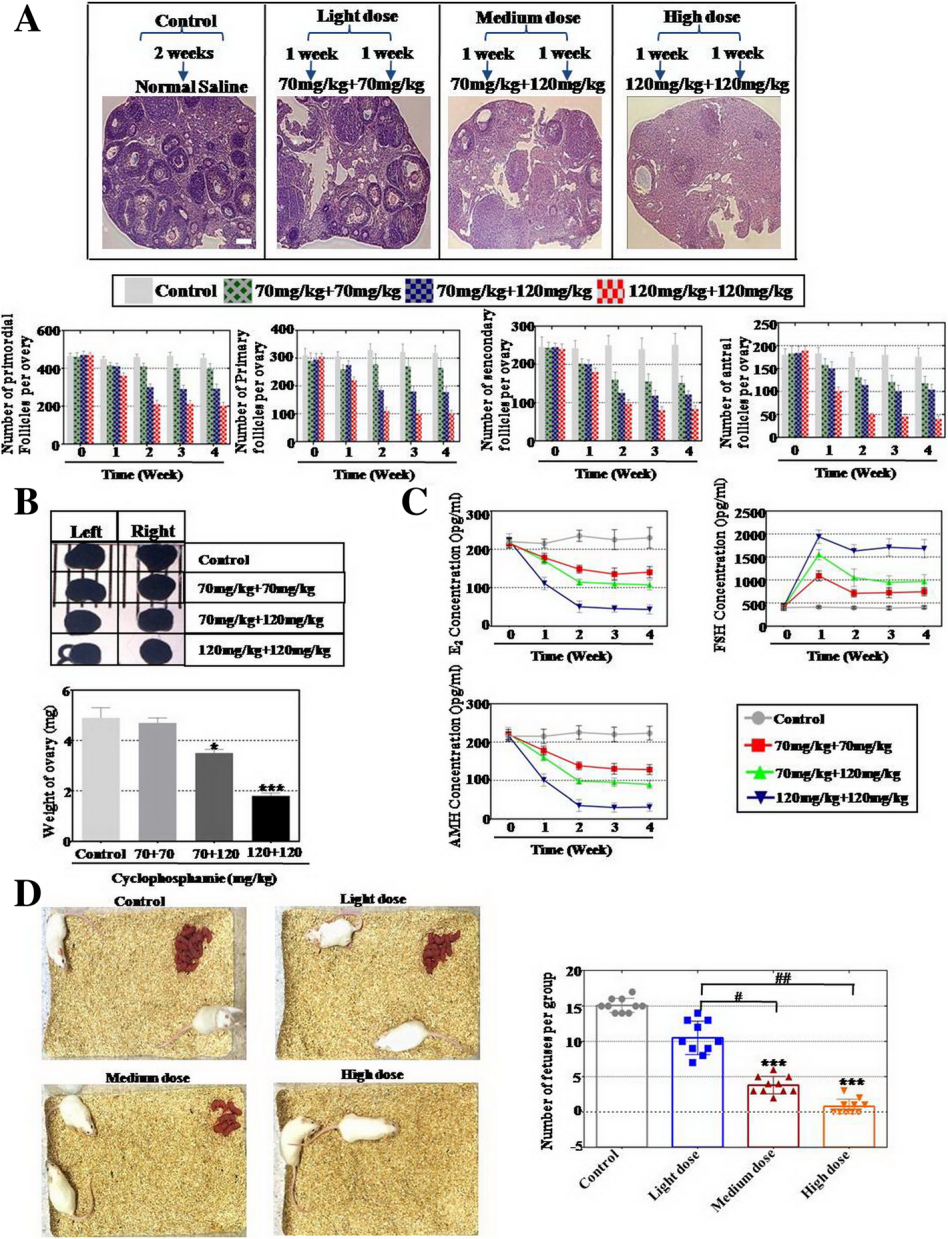
Established mice model with different degrees of POA. **a** Numbers of four stages of follicles (primordial, primary, secondary, and antral follicles) counted from 0 to 4 weeks after different doses of CTX treatment. **b** Weight of the ovary measured after different doses of CTX treatment. **c** Serum hormone levels of E2, AMH, and FSH measured by ELISA after different doses of CTX treatment. **d** Litter size from the mice model with different degrees of POA. All experiments were carried out three times. *Error bars* indicate SD. *p < 0.05, ***p < 0.001, compared to control group; #*p* < 0.05, ##*p* < 0.01, compared with light-dose group

To evaluate optimal effects between hAMSCs and hAECs to treat POA, we employed graded concentrations of CTX to treat female mice. According to the different concentrations we sorted three groups to treat, respectively the light-dose, medium-dose, and high-dose CTX groups. HE staining was used to assess the number of follicles at four stages during 4 weeks, respectively primordial, primary, secondary, and antral follicles. Our results demonstrated that the number of secondary and antral follicles decreased slightly to 60% and 67% separately at week 4 in the light-dose CTX group compared to the control group (Fig. 1a). The medium-dose CTX group showed an inhibition effect for follicles gradually at the four stages. Our results demonstrated that the numbers of secondary and antral follicles were decreased to 48% and 59% respectively after treatment for 4 weeks compared to the control group (Fig. 1a). The high-dose CTX group showed the most dominant effects to curb follicle numbers at the four stages. Results showed that the number of secondary and antral follicles were significantly decreased to 33% and 23% respectively at week 4 compared to the control group (Fig. 1a). In addition, we also tested the weight of the ovary after CTX treatment; assay results showed that the weight of the ovary almost did not change in the light-dose CTX group, but in the medium-dose and high-dose groups ovarian weight decreased significantly (Fig. 1b). Plasma samples from mice were collected and the ELISA method was employed to investigate the change of hormone levels (E2, FSH, and AMH) in the mice model. The levels of E2 and AMH were significantly lower in the high-dose CTX group than that in light-dose and medium-dose CTX groups. Simultaneously, the level of FSH was significantly higher in the high-dose CTX group than that in the light-dose and medium-dose CTX group compared to the normal healthy mice (Fig. 1c). After mice were treated with different CTX doses, the mice model and the control group were housed in the same cages as male mice to promote natural mating separately. The average number of offspring in the light-dose CTX group was lower (10.5 offspring) than that in the control group of saline-injected mice (15.1 offspring). The average number of offspring in the medium-dose CTX group was much lower (3.8 offspring) than in the control group. However, almost no offspring was found in the high-dose CTX group (Fig. 1d).

As a whole, a mice model with different levels of POA was established successfully by CTX treatment.

### hAMSCs restored ovary function more powerfully than hAECs in the high-dose CTX treatment mice

**Fig. 2:**
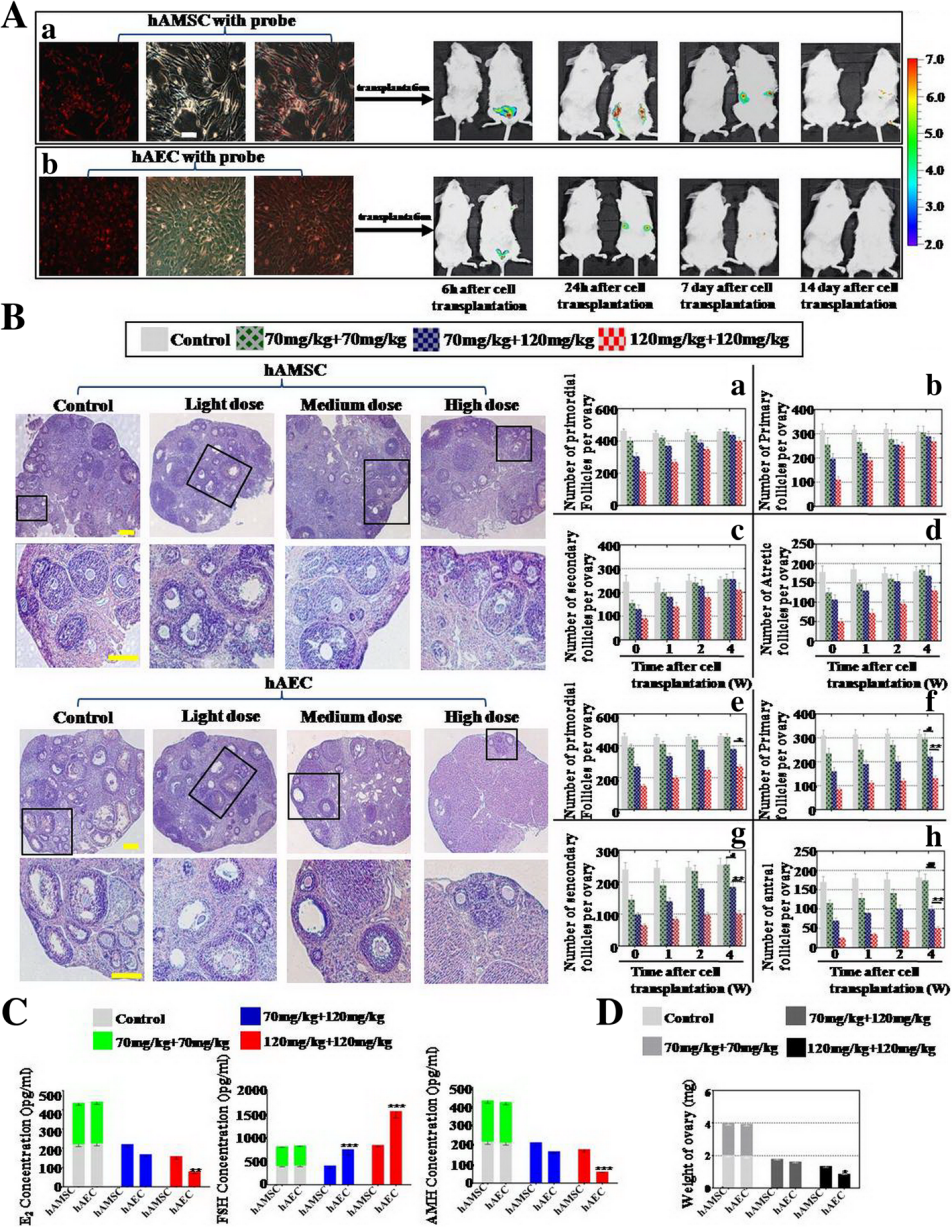
hAMSCs improved the function of the ovary more forcefully than hAECs. **a** Grafted (*a*) hAMSCs and (*b*) hAECs detected in vivo separately. Sterilized mice after tail-vein cell transplantation detected by live imaging for the identification of Dil-labeled cells in vivo. **b** Number of four-period follicles counted during 4 weeks after (*a–d*) hAMSC and (*e–h*) hAEC transplantation respectively. **c** Levels of E2, AMH, and FSH measured by ELISA at week 4 after hAMSC and hAEC transplantation respectively. **d** In the high-dose group, the weight of the ovary after hAEC transplantation was significantly lower than after hAMSC transplantation in the fourth week. All experiments were carried out three times. *Error bars* indicate SD. *p < 0.05, **p < 0.01,***p < 0.001, compared with medium-dose group; #*p* < 0.05, ##*p* < 0.01, compared with light-dose group. *hAEC* human amniotic epithelial cell, *hAMSC* human amniotic mesenchymal stem cell

**Fig. 3:**
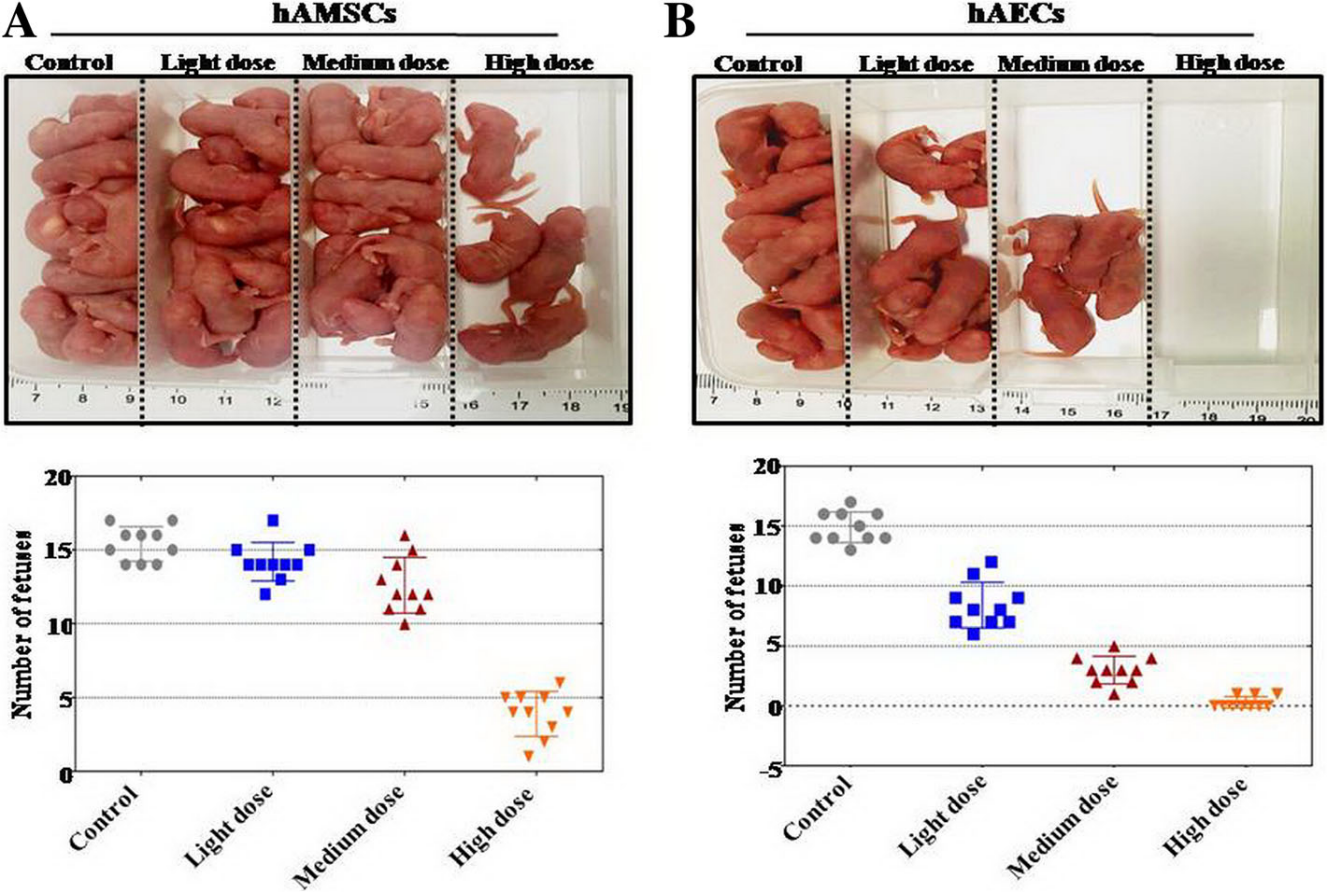
hAMSCs increased the number of offspring more than hAECs in the medium-dose and high-dose treated mice model. **a** Litter sizes counted after hAMSC transplantation into the mice model with different levels of ovarian aging. **b** Litter sizes counted after hAEC transplantation into the mice model with different levels of ovarian aging. *hAEC* human amniotic epithelial cell, *hAMSC* human amniotic mesenchymal stem cell

To investigate the distinct effects of hAMSCs and hAECs to restore ovary function, two kinds of cell lines were established that derived from hAM of spontaneous-conception Chinese woman. FACS was used to assess the characterization of hAMSCs and hAECs. Our results exhibited that cell surface markers (CD105, CD29, CD44, CD73, and C90) were highly expressed and two surface markers (CD34 and CD45) were rarely expressed in hAMSCs (Additional file 1: Figure S1A). hAMSCs were multipotent, as indicated by their ability to differentiate into adipocytes, chondroblasts, and osteoblasts (Additional file 1: Figure S1B). FACS assay results also indicated that cell surface markers (EpCam, CD73, and CD166) were highly expressed and two surface markers (CD44 and CD105) were rarely expressed in hAECs (Additional file 1: Figure S1C). To elucidate the stability of cells that homed in vivo after tail-vein injection, high-dose CTX-treated mice were screened by live imaging to identify Dil (red fluorescence)-labeled cells tracking from 6 h to 14 days after cell transplantation. As shown in Fig. 2Aa, the Dil-labeled hAMSCs first entered the pelvic organs at 6 h, and then migrated to the ovary from 24 h to 7 days. The signal was hardly detected at day 14. Our results also showed that the Dil-labeled hAECs first entered the pelvic organs at 6 h, and migrated to the ovary at 24 h. However, the signal was difficult to detect at day 7 after cell transplantation (Fig. 2Ab). HE-stained ovarian tissues showed that hAMSCs and hAECs have the ability to restore follicle numbers to the normal level in the light-dose CTX group in the four periods (Fig. 2b). In the medium-dose CTX group, our results exhibited that hAMSCs recovered follicle numbers to the normal level at week 4 (Fig. 2Ba–Bd). Differently, follicle numbers during the four periods were partly resilient to the normal level (84% primordial follicles, 70% primary follicles, 72% secondary follicles, 35% antral follicles) after hAEC transplantation at week 4 compared to that of the control group (Fig. 2Be–Bh). In the high-dose CTX group, hAMSCs could restore follicle numbers to 87% primordial follicles, 86% primary follicles, 71% secondary follicles, and 61% antral follicles at week 4 compared to that of the control group (Fig. 2Ba–Bd). However, the HE assay manifested that hAECs just recovered follicle numbers to 58% primordial follicles, 41% primary follicles, 40% secondary follicles, and 28% antral follicles compared to that of the control group in the fourth week (Fig. 2Be–Bh). The hormone level of plasma and the weight of ovaries in each group were determined. The ELISA test results revealed that there was no statistically significant difference in E2, FSH, and AMH levels after hAMSCs and hAECs were transplanted into the light-dose CTX group. In the high-dose CTX group, hAMSCs rescued the level of E2 (73%) and AMH (79%) more powerfully than hAECs (36% E2 and 29% AMH), and then the level of FSH decreased more in the hAMSC-transplanted group (211%) than hAECs (382%) compared to the control group (Fig. 2c). There was almost no statistically significant difference of the ovarian weight between light-dose and medium-dose CTX groups after hAEC and hAMSC transplantation separately. However, in the high-dose CTX group the ovarian weight was increased obviously after hAMSC transplantation compared to hAECs (Fig. 2d). To assess the distinct influence of hAMSC and hAEC transplantation on fertility, cell-transplanted mice were mated with normal male mice in order to prove fertilizing ability. The total number of pregnancies per group and pups per pregnancy were recorded. Light-dose CTX-treated female mice that underwent hAMSC transplantation had more pups (average number = 14.2) than hAECs (average number = 8.4). After hAMSC transplantation, the average number of offspring in the medium-dose and high-dose CTX groups was significantly higher (12.6 and 3.9 offspring) than in the hAEC-transplanted group (3.0 and 0.3 offspring) (Fig. 3a, b).

In summary, hAMSCs exhibited more powerful ability to restore ovarian function than hAECs.

### hAMSCs revealed more powerful ability to improve the proliferation rate of patients’ human ovarian granular cells (hGCs) than hAECs

**Fig. 4:**
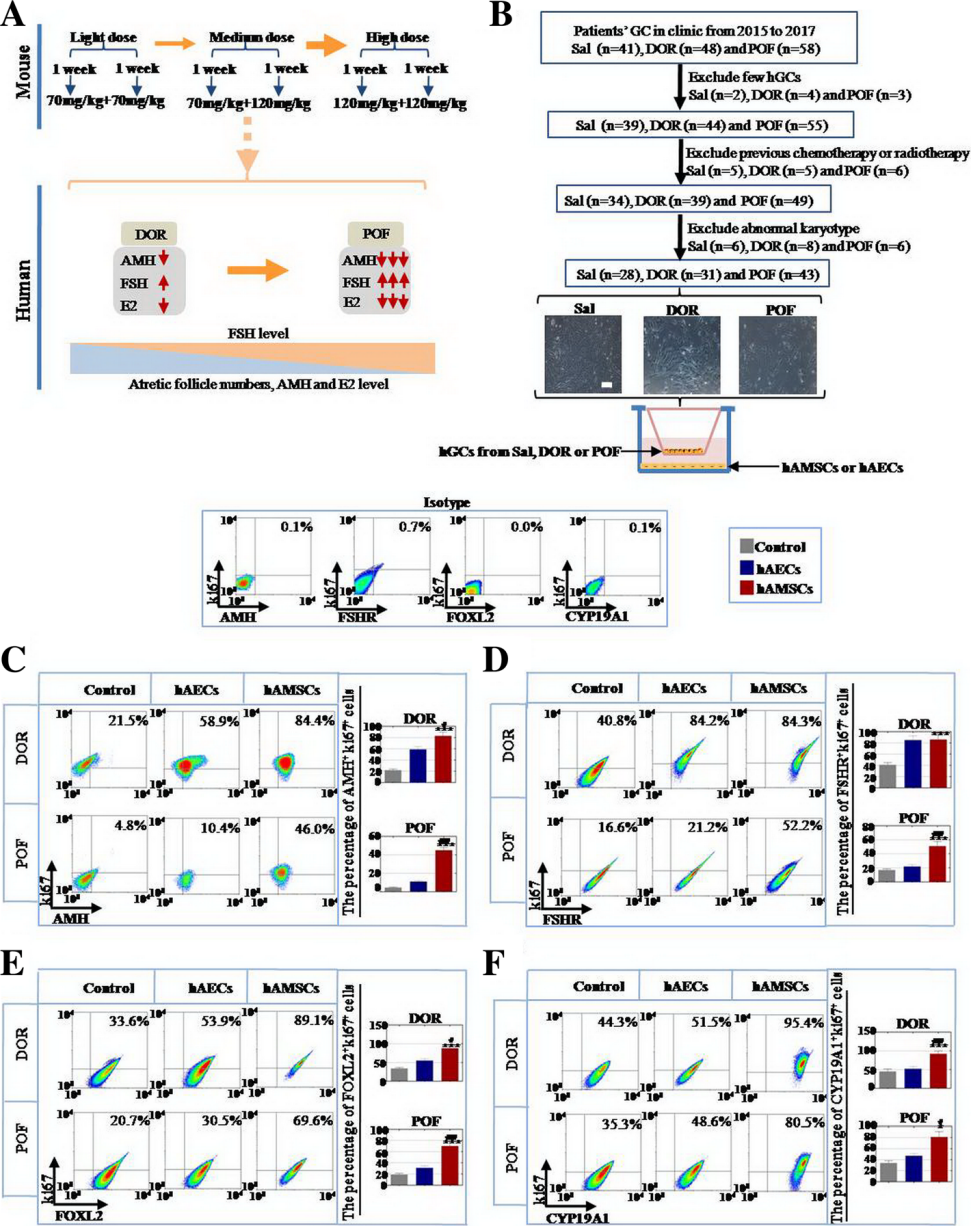
hAMSCs improved the proliferation rate of hGCs and upregulated the expression of hGC markers more forcefully than hAECs. **a** Schematic diagram of different degrees of ovarian aging mice model and patients. **b** Schematic overview of hGC filtered procedures. **c** Expression levels of ki67^+^FSHR^+^ hGCs tested after coculture with hAECs and hAMSCs respectively. **d** Number of ki67^+^AMH^+^ hGCs evaluated after coculture with hAECs and hAMSCs respectively. **e** Expression level of ki67^+^FOXL2^+^ hGCs tested after coculture with hAECs and hAMSCs respectively. **f** Number of ki67^+^CYP19A1^+^ hGCs evaluated after coculture with hAECs and hAMSCs respectively. Experiments were carried out after 7 days of coculture, *n* = 3. *Error bars* indicate SD. *p < 0.05, ***p < 0.001, compared with control group; #*p* < 0.05, ##*p* < 0.01, compared with hAEC group. *DOR* decreased ovarian reserve, *POF* premature ovarian failure, *Sal* saline, *hGC* human ovarian granulosa cell, *hAEC* human amniotic epithelial cell, *hAMSC* human amniotic mesenchymal stem cell

To investigate the therapy effects of hAMSCs and hAECs on different-level POA patients in the preclinical stage, we classified the POA patients into two groups from light to serious ovarian aging evaluated by the levels of E2, AMH, and FSH and antral follicle numbers: respectively DOR and POF. This kind of classification corresponded to light-dose, medium-dose, and high-dose CTX-treated mice groups (Fig. 4a). After filtration, we collected hGCs from TO, DOR, and POF patients in our reproductive center to examine the effects of cell proliferation after coculture with hAMSCs and hAECs respectively (Fig. 4b). Ki67 antibody (a cell proliferation marker) and four hGC markers (AMH, FSHR, FOXL2, and CYP19A1) were used to estimate the different effects between hAMSCs and hAECs by FACS analysis. Our results showed that hAMSCs increased ki67^+^AMH^+^ cell numbers more in the DOR and POF groups respectively (83% and 45%) than in the hAEC cocultured group (59% and 11%) compared to that of the control group (22% and 4.5%) (Fig. 4c). In Fig. 4d, FACS assay results demonstrated that hAMSCs increased ki67^+^FSHR^+^ cell numbers more in the POF group (51%) than in the hAEC cocultured group (22%) compared to that of the control group (17%), but no significant difference was detected between hAMSCs and hAECs cocultured with hGCs respectively in the DOR group. Our results revealed that hAMSCs increased ki67^+^FOLX2^+^ cell numbers more in the DOR and POF groups (88% and 70%) than in the hAEC cocultured group (55% and 31%) compared to that of the control group (34% and 19%) (Fig. 4e). In addition, FACS assay results manifested that hAMSCs raised ki67^+^CYP19A1^+^ cell numbers more in the DOR and POF groups separately (92% and 81%) than in the hAEC cocultured group (52% and 47%) compared to that of the control group (45% and 34%) (Fig. 4f).

In summary, hAECs exhibited less recovery effects for hGCs than hAMSCs, especially in the POF group.

### hAECs showed less immune rejection in patients’ PBMCs than hAMSCs

**Fig. 5:**
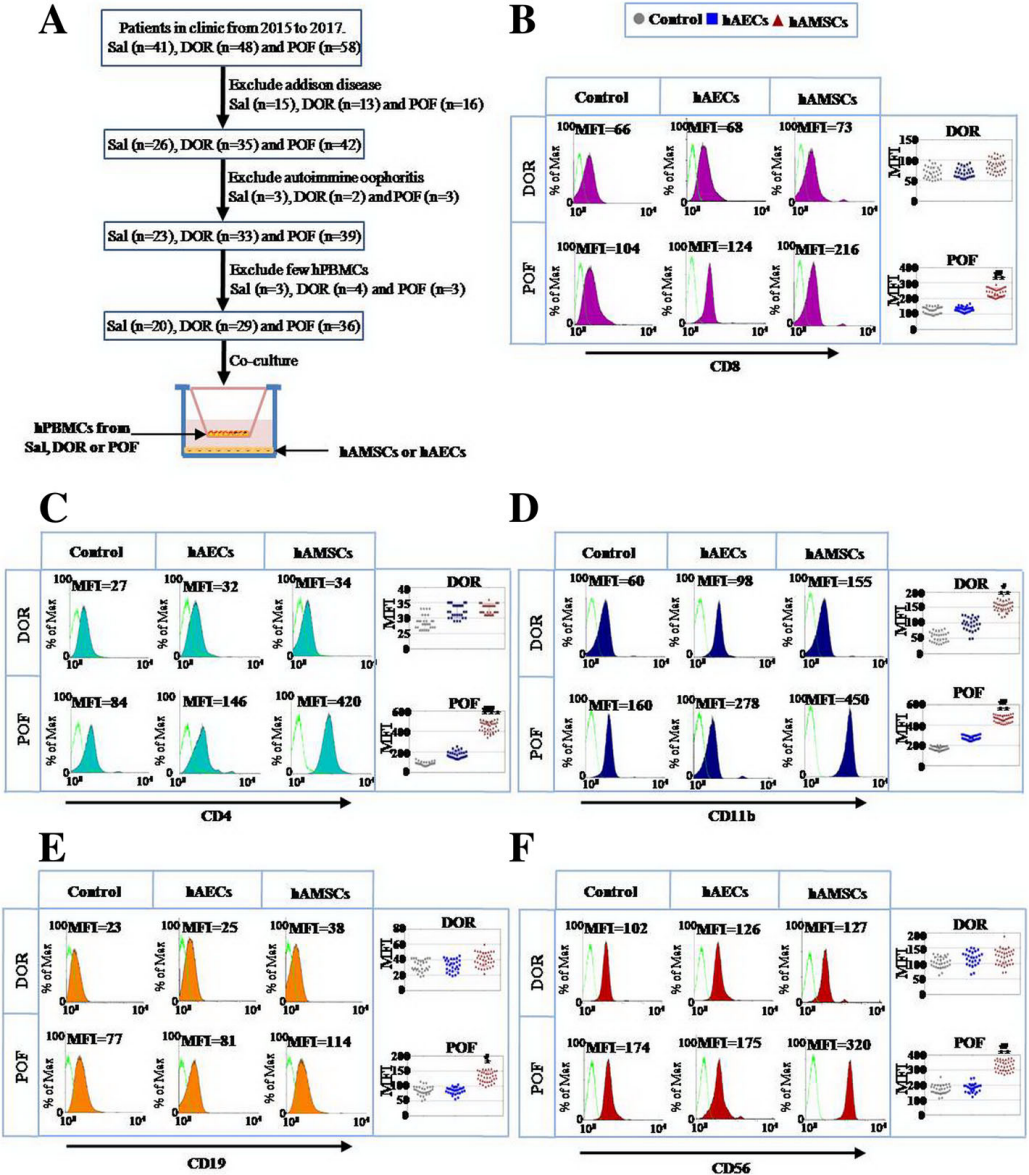
hAMSCs upregulated the expression level of immune molecules in hPBMCs more forcefully than hAECs. **a** Schematic overview of hPBMC filter procedures. **b** Expression level of CD8 in hPBMCs evaluated by FACS after coculture with hAECs and hAMSCs respectively. **c** Expression level of CD4 in hPBMCs evaluated by FACS after coculture with hAECs and hAMSCs respectively. **d** Expression level of CD11b in hPBMCs evaluated by FACS after coculture with hAECs and hAMSCs respectively. **e** Expression level of CD19 in hPBMCs evaluated by FACS after coculture with hAECs and hAMSCs respectively. **f** Expression level of CD56 in hPBMCs evaluated by FACS after coculture with hAECs and hAMSCs respectively. Experiments were carried out after 7 days of coculture, *n* = 3. *Error bars* indicate SD. *p < 0.05, **p < 0.01, ***p < 0.001, compared with control group; #*p* < 0.05, ##*p* < 0.01, compared with hAEC group. *Sal* saline, *DOR* decreased ovarian reserve, *POF* premature ovarian failure, *hPBMC* human peripheral blood mononuclear cell, *hAEC* human amniotic epithelial cell, *hAMSC* human amniotic mesenchymal stem cell, *​MFI* mean fluorescence intensity

To determine the immune rejection of hAMSCs and hAECs in the preclinical stage, hPBMCs from TO, DOR, and POF patients were cocultured with hAMSCs and hAECs respectively. The expression levels of immune molecules in hPBMCs were tested by FACS, such as CD4^+^ cells (Th cells), CD8^+^ cells (cytotoxic T cells), CD11b^+^ cells (macrophages/monocytes), CD19^+^ cells (B cells), and CD56^+^cells (natural killer cells) (Fig. 5a). Interestingly, our results demonstrated that hAMSCs increased the expression of CD8^+^ cells more prominently to 193% than hAECs to 107% in the POF group (Fig. 5b). The FACS assay exhibited similar results for CD4^+^ cells; hAMSCs increased the expression in CD4^+^ cells to 115% and 515% in the DOR and POF groups respectively; hAECs promoted the expression of CD4^+^ cells to 112% and 202% in the DOR and POF groups compared to that of the control group (Fig. 5c). The expression level of CD11b^+^ cells was significantly elevated to 284% and 270% in the DOR and POF groups after hAMSCs were cocultured with hPBMCs separately; the expression level of CD11b^+^ cells was elevated slightly to 176% and 160% in the DOR and POF groups after coculture with hAECs respectively compared to that of the control group (Fig. 5d). In the hAMSC cocultured group, the expression level of CD19^+^ cells was heightened to 126% and 155% in the DOR and POF groups respectively; in the hAEC cocultured group, the expression level of CD19^+^ cells was raised slightly to 101% and 100% in the DOR and POF groups respectively compared to that of the control group (Fig. 5e). The expression level of CD56^+^ cells was significantly increased in response to 119% (DOR) and 191% (POF) in the hAMSC cocultured group, and the expression level of CD56^+^ cells was almost not changed in response to 114% (DOR) and 105% (POF) respectively in the hAEC cocultured group (Fig. 5f).

In summary, hAMSCs caused stronger immune rejection than hAECs, particularly in the POF group.

### hAMSCs exhibited more dominant cellular biological characteristics than hAECs

**Fig. 6:**
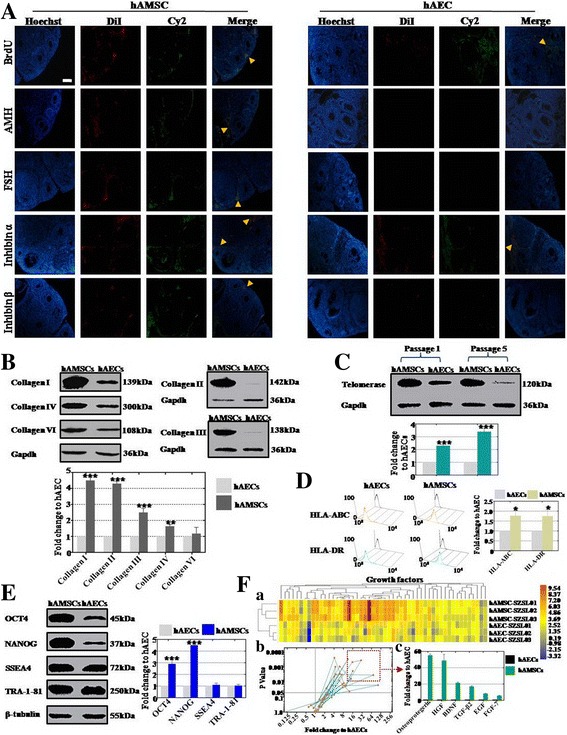
Distinction of cellular biological characteristics between hAMSCs and hAECs. **a** Expression of ovarian markers (AMH, FSH, inhibin α, and inhibin β) and proliferation marker (BrdU) in ovarian tissue measured after hAEC and hAMSC transplantation respectively. **b** Secretory level of collagen (I, II, III, and IV) from hAECs and hAMSCs estimated by western blot analysis respectively. **c** Activity of telomerase in hAECs and hAMSCs tested by western blot assay at passage 1 and 5 respectively. **d** Expression level of HLA-ABC and HLA-DR in hAECs and hAMSCs tested by FACS respectively. **e** Expression level of pluripotency markers (OCT4, NANOG, SSEA4, and TRA-1-81) in hAECs and hAMSCs measured by western blot analysis. **f** Growth factor derived from hAECs and hAMSCs estimated by antibody microarray respectively (*a–c*). fa heatmap exhibited the secretory level of growth facors between hAMSCs and hAECs; fb distribution of 52 growth factors were demonstrated after secretory level of hAMSCs compared to hAECs; fc in accordance with standard criteria of fold change ≥ 8 and statistical significance (p < 0.01), six growth factors were selected: osteoprotegerin, HGF, BDNF, TGF-β2, EGF, and FGF-7. All experiments were carried three times. *Error bars* indicate SD. *p < 0.05, **p < 0.01, ***p < 0.001, compared with hAECs group. *hAEC* human amniotic epithelial cell, *hAMSC* human amniotic mesenchymal stem cell

To investigate the different effects after hAMSC and hAEC transplantation into high-dose CTX-treated mice, the expression level of GC markers (AMH, FSH, inhibin α, and inhibin β) and proliferation ability (BrdU) were tested. Immunofluorescence assay results showed that Dil-hAMSCs and Dil-hAECs elevated the proliferation level in high-dose CTX-treated mice singly (Fig. 6a). Our results also revealed that Dil-hAMSCs promoted the expression levels of four GC biomarkers (AMH, FSH, inhibin α, and inhibin β) significantly in high-dose CTX-treated mice. However, Dil-hAECs just increased the expression level of one biomarker (inhibin α) in high-dose CTX-treated mice (Fig. 6a). The western blot method was employed to estimate various collagen secretion levels on hAMSCs and hAECs. Protein level results showed that the level of collagen secretion from hAMSCs was significantly higher than from hAECs, and reached 450% (collagen I), 430% (collagen II), 250% (collagen III), and 164% (collagen IV) on hAMSCs compared to that of hAECs, but not for collagen VI (118%) (Fig 6b). The protein level assay was also used to evaluate the expression level of telomerase activity between hAMSCs and hAECs. Results revealed that the expression level of telomerase from hAMSCs was more dominant than from hAECs, reaching 230% at passage 1 and 340% at passage 5 on hAMSCs compared to that of hAECs (Fig. 6c). Expression levels of HLA class I (A, B, C) and class II (DR) molecules on hAMSCs and hAECs were measured by FACS. Both HLA class I and class II molecules showed high expression to 177% and 175% on hAMSCs separately compared to that of hAECs (Fig. 6d). The pluripotent markers (OCT4, NANOG, SSEA4, and TRA-1-81) of hAMSCs and hAECs were tested by western blot analysis. Protein level results demonstrated that the expression levels of cell nuclei markers (OCT4 and NANOG) in hAMSCs were higher than hAECs, reaching 290% and 450% compared to that of hAECs. There were no changes in the protein expression level of cell membrane markers (SSEA4 and TRA-1-81) in hAMSCs compared to that of hAECs (Fig. 6e). In order to estimate the distinct cytokine production from hAMSCs and hAECs, supernatant was collected from three hAMS and hAE cell lines respectively that derived from three different pregnant women with spontaneous conception, and then a cytokine antibody array was utilized to evaluate this diversity. We classified the cytokine species from the antibody array results into four categories: growth factors (*n* = 52), chemotactic factors (*n* = 47), apoptosis factors (*n* = 19) and inflammatory factors (*n* = 55) group. Our analytical data elucidated that the number of growth factors (*n* = 46) from hAMSCs was higher than from hAECs (Fig. 6Fa). There were 22 growth factors secreted from hAMSCs that were significantly higher than hAECs (*p* < 0.05) (Fig. 6Fb). In accordance with standard criteria of fold change ≥ 8 and statistical significance (*p* < 0.01), six growth factors were selected: osteoprotegerin, HGF, BDNF, TGF-β2, EGF, and FGF-7 (Fig. 6Fc). Chemotactic factors were also detected by antibody array. Results showed that expression levels of fractional chemotactic factors (*n* = 29) from hAMSCs were much higher than in the hAEC group (Additional file 2: Figure S2Aa). There were 12 chemotactic factors from hAMSCs that were significantly higher than from hAECs (*p* < 0.05) (Additional file 2: Figure S2Ab). In accordance with the standard of fold change ≥ 8 and significant difference (*p* < 0.01), six chemotactic factors were selected: MCP-3, MCP-2, MIP-3-α, GCP-2, ENA-78, and LIF (Additional file 2: Figure S2Ac). Differently, the number of apoptosis factors from hAECs (*n* = 6, *p* < 0.05) was little more than that from hAMSCs (Additional file 2: Figure S2Ba, Bb). Meanwhile, no factors were selected according to the criterion of fold change > 8 and significant difference (*p* < 0.01). Then, our antibody array exhibited that there was no significant difference for inflammatory factors between hAMSCs and hAECs (Additional file 2: Figure S2Ca, Cb).

In summary, hAMSCs revealed more powerful cellular biological characteristics than hAECs, except for the expression level of immune molecules.

## Discussion

A variety of stem cells has been used to restore chemotherapy-induced ovarian failure, such as ADSCs, hAECs, human amniotic fluid stem cells, and human menstrual blood stem cells [14–16, 19]. However, understanding of how to evaluate the different effects of adult stem cells in curing POA and how to choose stem cells in clinical application is still lacking.

Many previous studies employed a single dose of CTX to inhibit ovarian function [19]; it is hard to investigate the effects of stem cell therapy for different degrees of POA. Therefore, in order to assess the different therapy effects between hAMSCs and hAECs, we successfully established a mouse model with different degrees of ovarian aging by combining doses of CTX. In this mice model, we observed that the number of follicles, level of hormones, weight of ovaries, and number of offspring were decreased significantly dose-dependently (Fig. 1). Our chemotherapy protocol is also supported by a recent study indicating that a different degree of male infertility mouse model was established after injection of busulfan with different doses [20]. Subsequently, hAMSCs and hAECs were transplanted into this model separately. Our results demonstrate that hAMSCs could restore ovarian function more forcefully than hAECs, especially in the high-dose CTX mice model group (Fig. 2). The present results hint that hAMSCs may be perceived as a more suitable cell resource in curing POA compared to hAECs.

The ultimate goal of research is to treat disease. To bridge the bench-to-bedside gap, preclinical efficacy of hAMSCs and hAECs for treating DOR and POF disease were evaluated; both of these cells were cocultured with hGCs derived from DOR and POF patients. Our findings indicated that hAMSCs increased the proliferation rate of hGCs more effectively than hAECs (Fig. 4). In the present study, the possible reason for the higher therapeutic activity of hAMSCs could be related to enormous secretion of collagen (Fig. 6B). In the meantime, we have found that activation of telomerase in hAMSCs was higher than in hAECs (Fig. 6C). These current findings are consistent with results of a previous study that collagen could maintain longer engraftment and survival of the cells in vivo [21]. A previous study revealed that the paracrine effect may play a key role in restoration of ovarian function [22]. In the present study, we found that hAMSCs excreted a much larger number of growth factors than hAECs (Fig. 6F). Moreover, the distinction of the therapeutic effect may be attributed to the fact that hAMSCs exhibited a higher expression of transcription factor OCT4 and NANOG than hAECs (Fig. 6E). These findings are similar to previous studies that upregulation of NANOG could improve production of cytokines via the JAK/STAT pathway [23, 24].

In addition, assessment of immune rejection is a necessary process for a preclinical trial [25]. Thereby, to evaluate the preclinical safety of hAMSCs and hAECs for treating DOR and POF patients, both of these cells were cocultured with hPBMCs respectively. Our study indicated that hAMSCs induced a greater immune response in hPBMCs than hAECs (Fig. 5). In the present research, one possible reason for such a distinction is that immune molecules were more strongly expressed in hAMSCs than in hAECs (Fig. 6d). Another possible reason for immune response could be related to a larger expression of OCT4 and NANOG in hAMSCs (Fig. 6e). This upregulated expression may explain the reason for initiating immune rejection in vivo. These findings are consistent with those reported previously and also support the idea that overexpression of transcription factor could induce immune rejection [26].

## Conclusions

This study is the first to assess the different therapy potential between hAMSCs and hAECs. Meanwhile, our findings indicated that hAMSCs are a more effective cell type to improve ovarian function than hAECs. Furthermore, our present study revealed that cellular biological characteristics of hAMSCs are superior to hAECs, except for immunological rejection. This discovery has important implications for understanding that cellular biological characteristics play a pivotal role in the distinction of stem cell therapy effects. Additionally, this study suggests that hAECs were suitable only for DOR disease. Meanwhile, a combination of hAMSCs and immunosuppressant was considered to treat POF disease better.

## Additional files

Additional file 1: Figure S1. is showing characterization of hAMSCs and hAECs tested. (**A**) Phenotype of CD105, CD29, CD44, CD73, CD90, CD34, and CD45 in hAMSCs detected by flow cytometry. (**B**) hAECs differentiate into adipocytes (Oil Red), osteoblasts (Alizarin red) and chondroblasts (Alcian blue) under standard in-vitro differentiating conditions. *Scale bars* = 10 μm. (**C**) Expression level of EpCam, CD44, CD73, CD105, and CD166 in hAECs detected by flow cytometry. (TIF 6049 kb)

Additional file 2: Figure S2. is showing distinction of cytokine levels between hAMSCs and hAECs. (**A**) Distinction of chemotactic factor levels between hAMSCs and hAECs. (**B**) Difference of apoptosis factor levels between hAMSCs and hAECs. (**C**) Difference of inflammatory factor levels between hAMSCs and hAECs. (TIF 8400 kb)
