# Supplementary figures and images for: Different therapeutic effects of cells derived from human amniotic membrane on premature ovarian aging depend on distinct cellular biological characteristics
Source: Stem Cell Res Ther. 2017 Jul 27;8:173. doi: 10.1186/s13287-017-0613-3 (PMC5530953; doi:10.1186/s13287-017-0613-3)

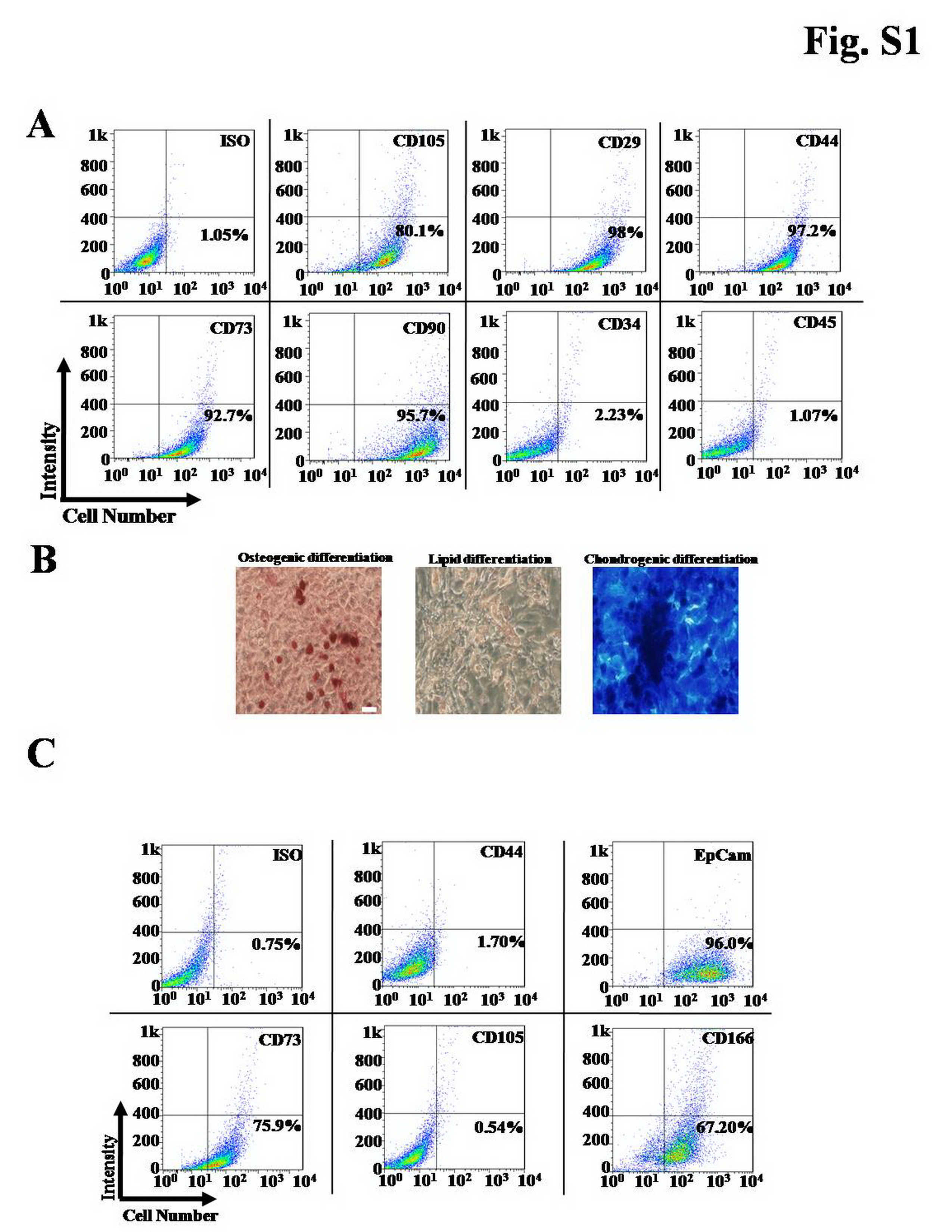

Supplement: Supplementary file 1 — is showing characterization of hAMSCs and hAECs tested. (A) Phenotype of CD105, CD29, CD44, CD73, CD90, CD34, and CD45 in hAMSCs detected by flow cytometry. (B) hAECs differentiate into adipocytes (Oil Red), osteoblasts (Alizarin red) and chondroblasts (Alcian blue) under standard in-vitro differentiating conditions. Scale bars = 10 μm. (C) Expression level of EpCam, CD44, CD73, CD105, and CD166 in hAECs detected by flow cytometry. (TIF 6049 kb) [file 13287_2017_613_MOESM1_ESM.tif]

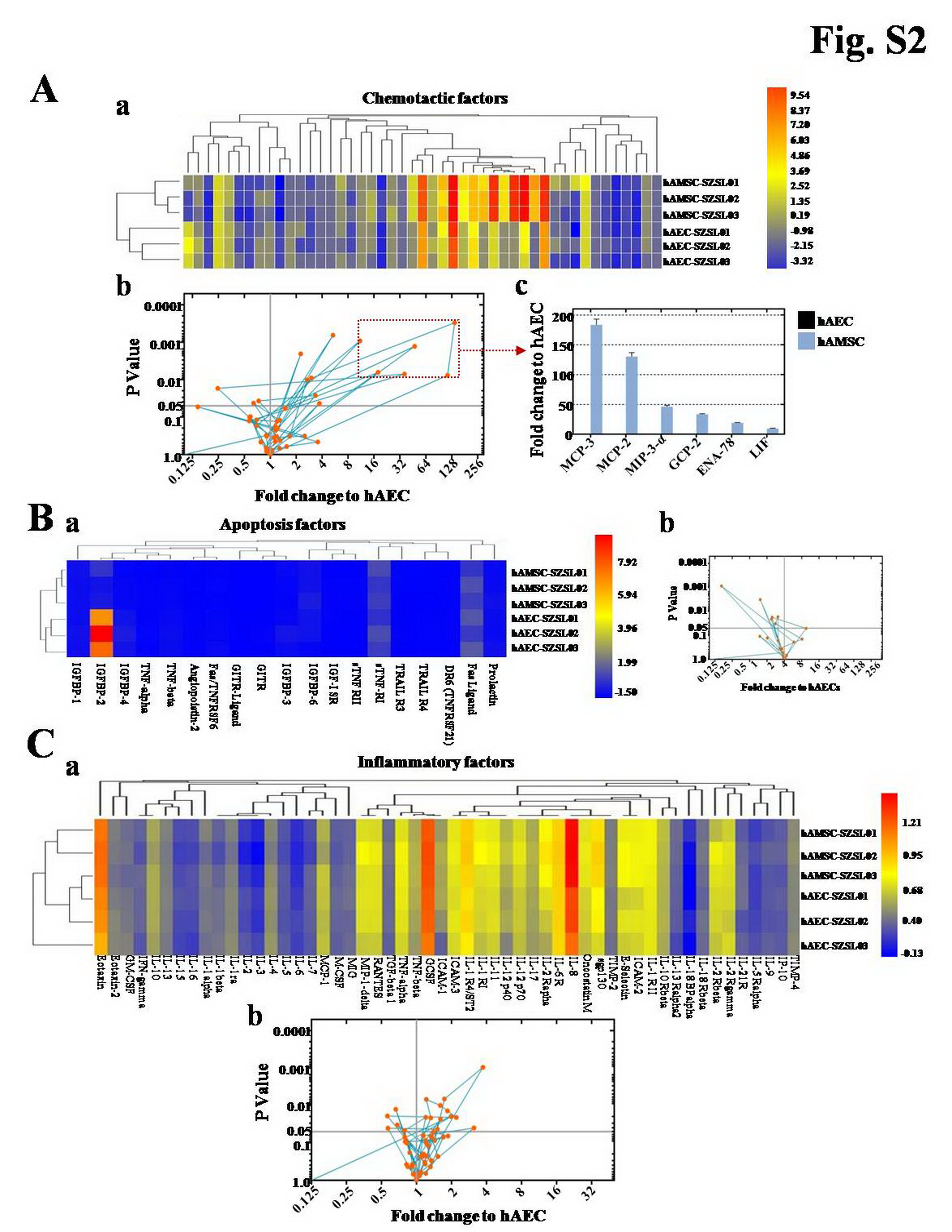

Supplement: Supplementary file 2 — is showing distinction of cytokine levels between hAMSCs and hAECs. (A) Distinction of chemotactic factor levels between hAMSCs and hAECs. (B) Difference of apoptosis factor levels between hAMSCs and hAECs. (C) Difference of inflammatory factor levels between hAMSCs and hAECs. (TIF 8400 kb) [file 13287_2017_613_MOESM2_ESM.tif]
